# The expression of hTR and hTERT in human breast cancer: correlation with clinico-pathological parameters

**DOI:** 10.1186/1477-7800-3-20

**Published:** 2006-08-22

**Authors:** Saied Hosseini-Asl, Morteza Atri, Mohammad H Modarressi, Mohamed Salhab, Kefah Mokbel, Parvin Mehdipour

**Affiliations:** 1 Department of Medical Genetics, School of Medicine, Tehran University of Medical Sciences, Tehran, IR, Iran; 2 Cancer Institute, Tehran University of Medical Sciences/Day General Hospital, Tehran, IR, Iran; 3 St George's Hospital, London, SW17 0QT, UK

## Abstract

**Background:**

Telomerase is a ribonucleoprotein enzyme that synthesises telomeres after cell division and maintains chromosomal stability leading to cellular immortalization. Telomerase has been associated with negative prognostic indicators in some studies. The present study aims to detect any association between telomerase sub-units: hTERT and hTR and the prognostic indicators including tumour's size and grade, nodal status and patient's age.

**Methods:**

Tumour samples from 46 patients with primary invasive breast cancer and 3 patients with benign tumours were collected. RT-PCR analysis was used for the detection of hTR, hTERT, and PGM1 (as a housekeeping) genes expression.

**Results:**

The expression of hTR and hTERT was found in 31(67.4%) and 38 (82.6%) samples respectively. We observed a significant association between hTR gene expression and younger age at diagnosis (p = 0.019) when comparing patients ≤ 40 years with those who are older than 40 years. None of the benign tumours expressed hTR gene. However, the expression of hTERT gene was revealed in 2 samples.

No significant association between hTR and hTERT expression and tumour's grade, stage and nodal status was seen.

**Conclusion:**

The expression of hTR and hTERT seems to be independent of tumour's stage. hTR expression probably plays a greater role in mammary tumourogenesis in younger women (≤ 40 years) and this may have therapeutic implications in the context of hTR targeting strategies.

## Background

Telomerase is an RNA dependant DNA polymerase, the function of which is to synthesise the repetitive nucleotide sequence (TTAGGG in humans) forming the telomeres at the end of chromosomes [1]. These telomeres form caps on the chromosomes that prevent fusion of chromosomal ends during cell division. The DNA polymerase is unable to fully replicate the ends of linear DNA and genetic material is lost and this can result in chromosomal instability and cellular senescence. Without telomerase activity, each round of nuclear division results in shortening of telomeres and reaching a critical length seems to trigger off cellular apoptosis. Therefore, telomerase activity seems to stabilize telomeres and to be responsible for compensating about 65 bp in eukaryotic chromosomal ends, thus, leading to cellular immortality [1-8] and, may therefore, be a critical step in carcinogenesis [9,11].

Telomerase is active in 70 – 90% of malignant tissues and many immortal cell lines, but most somatic cells have no detectable telomerase activity [5,10,11].

Human telomerase consists of an RNA subunit; human telomerase RNA (hTR) [12], a protein component (human telomerase associated protein 1 (hTEP1) [13] and the catalytic subunit hTERT (human telomerase reverse transcriptase) [14-16]. Of these subunits telomerase activity requires the presence of hTR, which is the RNA template for the telomeric repeat, and hTERT, which is the reverse transcriptase. Furthermore, it is reported that the induction of hEST2 mRNA expression is required for the telomerase activation that occurs during cellular immortalization and tumor progression. [17]. Several studies have shown that disrupting the function of telomerase RNA leads to progressive shortening of telomeres, suggesting that this component plays an essential role in telomerase function [18].

The genes coding for the RNA subunits of telomerase have been cloned from a wide variety of species [12,19] and have been shown to be essential for telomerase function in vivo [18,20-23]. In humans, genes coding for hTERT and hTR have been cloned and mapped to chromosomes 5p15.33 and 3q26.3 respectively [4,24].

Expression of hTERT was found to be at high levels in malignant tumors and cancer cell lines but not in normal tissues or telomerase-negative cell lines, and a strong correlation was found between hTERT expression and telomerase activity in breast cancer [25].

We previously reported that telomerase reactivation was significantly associated with advanced breast cancer stage, histopathological grade [26] and nodal metastasis with no significant association between telomerase activity and menopausal status, or tumor size [27]. Furthermore, we reported no significant association between tumour hTERT expression and patient's age, tumour size, grade, nodal metastasis, estrogen receptor (ER) positivity and lymphovascular (LVI) [28,29].

The aim of this study was to examine the expression of telomerase subunits hTERT and hTR and correlate them with different clinical and pathological parameters including tumour's size and grade, nodal status and patient's age in human breast cancer.

## Materials and methods

### Patients

Institutional guidelines including ethical approval and informed consent were followed. Samples from 46 patients with primary invasive breast cancer and 3 patients with benign breast lesions were studied. All patients were surgically treated at (Day General Hospital, Tehran, Iran) during 2004–2005. Breast tissues were collected and preserved by rapid freezing in liquid nitrogen immediately after surgical excision and then were stored at -70°C.

### RT-PCR analysis

Total RNA was isolated from samples using Tripure Isolation Reagent (Roche, Germany). The mixture of one microgram of total RNA, random hexamer and M-Mulv reverse transcriptase enzyme (Fermentas Co, Canada) was used to create cDNA for each sample, according to the manufacturer's protocol. In order to avoid he probable DNA contamination for RNA samples, the following stages were performed. We prepared a solution containing the same materials used for cDNA synthesis excluding reverse transcriptase enzyme (negative control 1). This product contains DNA only, with a new concentration similar to the cDNA products. However, in the PCR products of these samples, the presence or absence of any DNA contamination could be observed and detected.

In order to perform DNase treatment, 1 μg of total RNA was digested by DNase (Fermentas Co, Canada) according to the manufacturer's protocol. Half of DNase treated RNA sample was used to create cDNA. The remaining half of the sample contained all of the materials excluding the reverse transcriptase enzyme (negative control 2), in order to validate the accuracy of DNase treatment process.

The cDNA, DNase treated cDNA and control group samples (to evaluating the accuracy of DNase treatment) were amplified in a 25 μl reaction mixture containing 0.2 μM of each primers and 1U Taq DNA polymerase (Fermentas Co, Canada). For detecting the accuracy of RNA extraction and cDNA synthesis, phosphoglucomutase 1 (PGM1) housekeeping gene was used in RT-PCR performance. The sequence of oligonucleotides which were used for amplification of PGM1, hTR and hTERT genes is listed in table 1. Amplified products were subjected to electrophoresis in 2% agarose gels and were visualized with ethidium bromide.

**Table 1:** The oligonucleotide sequence of primers used for detecting expression of genes of interest, in the RT-PCR assay.

Oligonucleotide	Sequence	Amplified length	Annealing temperature
PGM1–1718	5'-TCCGACTGAGCGGCACTGGGAGTGC-3'	386bp	63°C
PGM1–2080	5'-TCCGACTGAGCGGCACTGGGAGTGC-3'		
hTR-F	5'-CGCCGTGCTTTTGCT CC-3'	316bp	63°C
hTR-R	5'-ACTCGCTCCGTTCCTCTTCC-3'		
hTERT–13187	5'-CGG AAG AGT GTC TGG AGC AA-3'	145bp	67°C
hTERT–15411	'5'-TCC AGA CTC CGC TTC ATC C-3'		

### Statistical analysis

The statistical analysis of the data was carried out by using the SPSS software package (SPSS Inc; Chicago, IL, USA; Version 11.5, 2003). The Pearson chi-square and Fisher Exact test were performed for the analysis of probable contributions. The significance levels were considered for results with P value lower than 0.05.

## Results

The mean age of patients with primary breast cancer at diagnosis was 47 years with a range of (28–71). 22.2% (10/45) of the patients were diagnosed at the age of 40 or younger.

**Figure 1:**
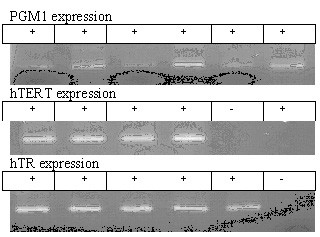
The electropherogram of the PGM1, hTR and hTERT genes RT-PCR

hTERT and hTR genes expression were detected in 38 (82.6%) and 31 (67.4%) breast cancers respectively (Figure 1) (Table 2). hTR gene was expressed in all cancers from patients aged ≤ 40 years (10/10) compared to 60 % (21/35) of patients aged > 40 years (p = 0.019). This significant observation was not seen with hTERT. Furthermore, there was a significant association between hTERT expression and hTR expression when comparing patients aged ≤ 40 with those who are > 40 years old. (p = 0.018).

**Table 2:** hTR and hTERT expression in 46 malignant samples

	hTR expression	hTERT expression
Positive	67.4% (31)	82.6 %(38)
Negative	32.6 %(15)	17.4% (8)

Tumour size range was (0.9–7.0 cm) with a mean of 2.67 cm. We observed no association between tumour size and expression of hTR and hTERT (Table 3) Moreover, no association was seen between hTR, hTERT expression and tumour's grade, stage, axillary node status and pathological type of the tumour.

**Table 3:** Correlation between hTR, hTERT and clinicopathological parameters

**Parameter**		**Freq**							**hTERT**^+ ^**samples**		
									
			**hTR expression**			**hTERT expression**			**hTR expression**		
			
			Neg.	Pos.	P	Neg.	Pos.	P	Neg.	Pos	P
**Age range**	≤40 >40	10 (22.2)* 35 (77.8)	0 14	10 21	**0.019**	0 8	10 27	0.168	0 11	10 16	**0.018**
**Size range**	≤2 cm	22 (47.8)	5	17	0.2	4	18	0.538	4	14	0.27
	2–5 cm	19 (41.3)	9	10		4	15		7	8	
	>5 cm	5 (10.8)	1	4		0	5		1	4	
**Pathol.**	IDC	43 (95.5)	15	28	0.593	8	35	0.798	12	23	0.602
	ILC	1 (2.2)	0	1		0	1		0	1	
	Muc.	1 (2.2)	0	1		0	1		0	1	
**Grade**	I	4 (9.8)	1	3	0.282	1	3	0.958	1	2	0.765
	II	16 (39)	3	13		3	13		3	10	
	III	21 (51.2)	9	12		4	17		6	11	
	Low	20 (48.8)	4	16	0.18	4	16	1	4	12	0.7
	High	20 (51.2)	9	12		4	17		6	11	
**ALN Inv.**	Positive	26 (57.8)	6	20	0.2	4	22	0.7	5	17	0.29
	Negative	19 (42.2)	8	11		4	15		6	9	
**Stage**	I	9 (30)	4	5	0.087	2	7	0.941	3	4	**0.045**
	IIA	7 (23.3)	2	5		2	5		1	4	
	IIB	7 (23.3)	5	2		1	6		5	1	
	IIIA	6 (20)	0	6		1	5		0	5	
	IIIB	1 (3.3)	0	1		0	1		0	1	

Finally, two of the benign breast lesion showed hTERT expression. However, none of them expressed hTR (Table 4).

**Table 4:** Telomerase subunits expression in 3 non-malignant samples

	Pathology	hTR expression	hTERT expression
1	Fibrocystic change	Negative	Positive
2	Fibrocystic change	Negative	Negative
3	Benign	Negative	Positive

## Discussion

It is well established that telomerase activity requires the presence of its subunits; hTR and hTERT. This study focuses on a new angle in the understanding of telomerase regulation in breast cancer. To our knowledge, this is the first study to examine the association between hTR and the prognostic factors in human breast cancer. We used the DNase treatment method for the detection of hTR gene expression in order to avoid DNA contamination in RNA extracts. Such contamination may result in false positive findings when RT-PCR technique is used alone in those genes that lack introns or contain pseudogene (such as GAPDH housekeeping gene). Previous studies did not perform the DNase treatment method prior to cDNA synthesis. This resulted in hTR being expressed in both cancer and benign cells alike, hence, very little attention was given to hTR role in the telomerase regulation and correlation with prognostic factors [30-39].

We found that benign breast lesions showed no expression of hTR. Such an observation agrees with other studies; Yashima et al showed that hTR expression in stromal cells, including those in fibroadenomas, was negative and increased hTR expression was observed in some foci of apocrine metaplasia and atypical hyperplasia [40]. Moreover, a multistage tumorigenesis study in transgenic mice has shown that the RNA component of telomerase is up-regulated in the first stages of tumorigenesis, even in precancerous lesions [41]. Therefore, up-regulation of hTR may be a predictive marker for invasive tumor development.

Our observation that hTR expression was associated with younger age (patients aged ≤ 40 years) is a very interesting one. This has implication regarding telomerase gene based therapy or cancer treatment strategies in young patients with breast cancer such as targeting the template region of hTR with anti hTR (such as oligonucleotides) which may inhibit cell telomerase activity and cell proliferation and can lead to a profound induction of programmed cell death [41,42]

We conclude that hTR expression probably plays a greater role in mammary tumourogenesis in younger women (≤ 40 Years.). Tumours in older patients may develop telomerase independent mechanisms for survival.
